# The Potential Health Benefits of the Ketogenic Diet: A Narrative Review

**DOI:** 10.3390/nu13051654

**Published:** 2021-05-13

**Authors:** Kathryn Dowis, Simran Banga

**Affiliations:** Department of Biology, Western Kentucky University, Bowling Green, KY 42101, USA; kathryn.dowis803@topper.wku.edu

**Keywords:** β-hydroxybutyrate (BHB), body mass index (BMI), type 1 diabetes, type 2 diabetes (T2D), hemoglobin A1c (HbA1c), visceral adipose tissue (VAT), cardiovascular disease (CVD), high-density lipoprotein (HDL), low-density lipoprotein (LDL), Apolipoprotein B (ApoB)

## Abstract

Considering the lack of a comprehensive, multi-faceted overview of the ketogenic diet (KD) in relation to health issues, we compiled the evidence related to the use of the ketogenic diet in relation to its impact on the microbiome, the epigenome, diabetes, weight loss, cardiovascular health, and cancer. The KD diet could potentially increase genetic diversity of the microbiome and increase the ratio of *Bacteroidetes* to *Firmicutes*. The epigenome might be positively affected by the KD since it creates a signaling molecule known as β-hydroxybutyrate (BHB). KD has helped patients with diabetes reduce their HbA1c and reduce the need for insulin. There is evidence to suggest that a KD can help with weight loss, visceral adiposity, and appetite control. The evidence also suggests that eating a high-fat diet improves lipid profiles by lowering low-density lipoprotein (LDL), increasing high-density lipoprotein (HDL), and lowering triglycerides (TG). Due to the Warburg effect, the KD is used as an adjuvant treatment to starve cancer cells, making them more vulnerable to chemotherapy and radiation. The potential positive impacts of a KD on each of these areas warrant further analysis, improved studies, and well-designed randomized controlled trials to further illuminate the therapeutic possibilities provided by this dietary intervention.

## 1. Introduction

Ketogenic diets have started to increase in popularity as doctors and researchers investigate the potential benefits. Nutritional ketosis, the aspirational endpoint of ketogenic diets, is achieved by restricting carbohydrate intake, moderating protein consumption, and increasing the number of calories obtained from fat [1]. Theoretically, this restriction of carbohydrates causes the body to switch from glucose metabolism as a primary means of energy production. This results in the use of ketone bodies from fat metabolism, a metabolic state where the body prefers to utilize fat as its primary fuel source. Recent studies utilizing Low-carbohydrate, High-fat (LCHF) diets, such as the ketogenic diet, show promise in helping patients lose weight, reverse the signs of metabolic syndrome, reduce, or eliminate insulin requirements for type II diabetics [2], reduce inflammation, improve epigenetic profiles, alter the microbiome, improve lipid profiles, supplement cancer treatments, and potentially increase longevity [3] and brain function. 

**Figure 1:**
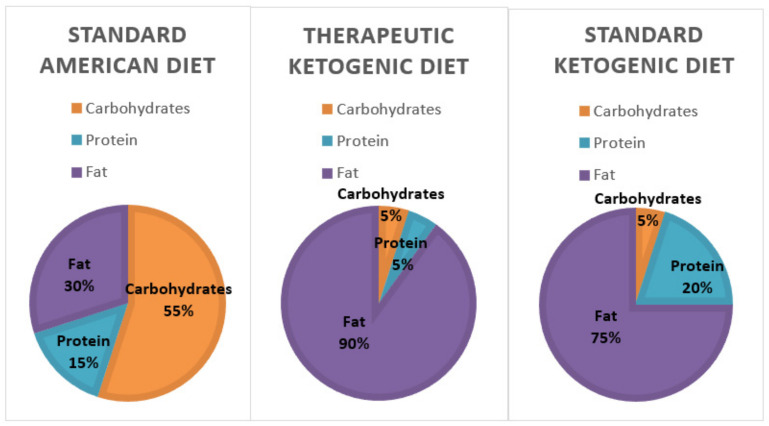
A comparison between the macronutrient breakdown of the standard American diet, therapeutic ketogenic diet, and the typical ketogenic diet. The therapeutic ketogenic diet is typically used in epilepsy and cancer treatments.

The number of Americans suffering from obesity, diabetes, and metabolic syndrome is on the rise. The markers of metabolic syndrome include an increase in abdominal adiposity, insulin resistance, elevated triglycerides, and hypertension [4,5]. All of these negative health markers increase the risk of cardiovascular disease, diabetes, stroke, and Alzheimer’s disease. According to WebMD, there are currently 27 million people with Type 2 diabetes and 86 million with pre-diabetes. In addition, the Centers of Disease Control and Prevention (CDC) also estimates that almost 40% of adults and around 20% of American children are obese [6,7]. Many researchers believe these diseases are a result of carbohydrate intolerance and insulin resistance. Thus, a diet that reduces the exposure to carbohydrates, including whole grains, might become a more logical recommendation for improving health [8]. In line with this, two dietary regimens, the standard ketogenic diet, and the therapeutic ketogenic diet (Figure 1), which restrict carbohydrate consumption to varying degrees are being studied for their health impacts. The therapeutic ketogenic diet, which severely restricts both carbohydrates and protein, is typically used in the treatment of epilepsy and cancer. However, the Dietary Guidelines for Americans suggests that between 45 and 65% of caloric intake should come from carbohydrates (Figure 1). If a person consumed 2000 calories per day that would equate to an average of 225–325 g of carbohydrates each day [9]. 

**Figure 2:**
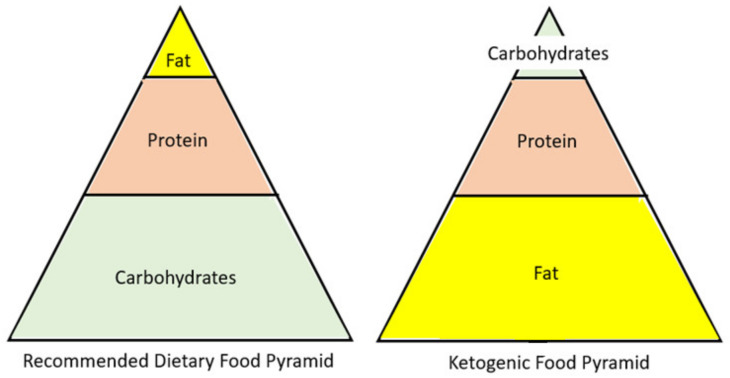
A visual comparison of the recommended dietary food pyramid, including major macromolecule components, to the ketogenic diet food pyramid.

One emerging diet that is becoming mainstream is a low-carb/high-fat diet. However, there is a difference between a low-carb and a low-carb ketogenic diet (LCKD). Ketosis is normally achieved through either fasting or carbohydrate restriction. It is important to clarify that a low-carb diet typically refers to a diet with an intake of 50 to 150 g of carbohydrate per day. However, although this is a lower amount of carbohydrates than the standard American diet, it is not low enough to enter nutritional ketosis. Only when a patient restricts carbohydrates to less than 50 g/day will the body be incapable of fueling the body by glucose and will switch to burning fat [10]. The ketogenic diet is a reversal of the current food pyramid supported by the dietary guidelines. Thus, instead of a diet rich in carbohydrates, it is high in fat (Figure 2). The resulting carbohydrate restriction lowers blood glucose levels, and the subsequent insulin changes will instruct the body to change from a state of storing fat to a state of fat oxidation [10]. Once fats are utilized as the primary fuel source in the liver, the production of ketone bodies begins, a process known as ketogenesis. During ketosis, three major ketone bodies are formed and utilized by the body for energy: acetone, acetoacetate, and β-hydroxybutyrate [11]. All cells that contain mitochondria can meet their energy demands with ketone bodies, including the brain and muscle. In addition, research suggests that β-hydroxybutyrate acts as a signal molecule and may play a role in suppressing appetite [12].

**Figure 3:**
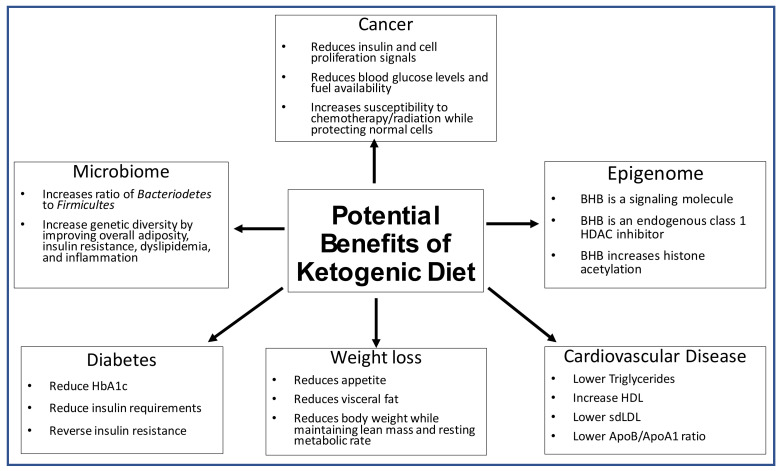
The potential therapeutic impacts of the ketogenic diet on the microbiome, epigenome, diabetes, weight loss and cardiovascular disease.

However, there is some heterogeneity in the available data. Thus, the aim of this review is to highlight the role the ketogenic diet has in altering the microbiome, epigenetics, weight loss, diabetes, cardiovascular disease, and cancer as summarized below (Figure 3).

## 2. The Effect of the Ketogenic Diet on the Microbiome

The microbiome consists of trillions of microscopic organisms in the human gastrointestinal tract. It comprises over 8000 different types of bacteria, viruses, and fungi living in a complex ecosystem [13]. Recent research suggests that the genetic make-up of a microbiome can be affected by lifestyle factors which include but are not limited to sleep, exercise, antibiotic use, and even diet. These bacteria can alter our response to different food sources because they differ in their ability to harvest energy from food, affecting the postprandial glucose response (PPGR) [13]. Since the controlling of glucose levels in the blood seems to reduce the risk of metabolic disease, diabetes, and obesity, this might be an innovative way to help reduce disease risk. A study conducted at the Weizmann Institute demonstrated that a mathematical algorithm could be used to determine an individual’s microbiome profile and predict their glycemic response to different types of foods [14]. Thus, the patients were able to change from stable blood glucose to unstable levels by simply eating the foods that the program predicted as good or bad based on their microbiome. Their initial results were confirmed by a repeat study at the Mayo Clinic with a different population [13]. It is important to note that the composition of the microbiome, which is believed to have a fundamental role in human health, is shaped predominantly by environmental factors. According to a study conducted by Rothschild et al. [15], the average heritability of the gut microbiome taxa is only 1.9%, while over 20% of variability was associated with diet and lifestyle. 

Thus, research into the complex interactions that exist between diet, the microbiome, and host metabolic rates have increased. A study exploring the benefits of prebiotic foods, such as inulin and oligosaccharides, observed an increase in the number of *Bifidobacteria* in the colon and the presence of other critical butyrate-producing bacteria [16]. Another study determined that the diversity of the gut microbiota was influenced more by a Westernized diet than by the body mass index of the subjects [17]. The patients who followed the Westernized diet showed an increase in *Firmicutes* and a decrease in *Bacteroidetes* in their microbiome, which are negative changes. A review article also reported positive changes in the gut microbiome and overall health in energy-restrictive diets or diets rich in fiber and vegetables [18]. Thus, people eating processed and bland food had reduced diversity of their microbiota, while people eating a diet rich in fruit and vegetables had increased diversity in their gut microbiota [19]. Moreover, gut biomes that lacked genetic diversity were related to overall adiposity, insulin resistance, dyslipidemia, and an inflammatory phenotype [20]. 

Discovering how the gut microbiota and diet interact and how this interaction is connected to overall health, is critical. It is important to determine whether new dietary changes, such as a ketogenic diet, will positively or negatively affect overall microbiome diversity and species make-up. Some research has found that whole grains play an important role in the development of a healthy microbiome and are necessary for good health [21]. Thus, a person consuming a ketogenic diet might not consume enough whole grains to maintain a healthy microbiome [12]. According to Adam-Perrot et al. [12] low-carb diets are at greater risk of being nutritionally inadequate by lacking in fiber, necessary vitamins, minerals, and iron. This idea is based on analysis of popular diets and food surveys conducted to determine nutrient intake while consuming varying levels of carbohydrates [22]. Thus, it is even more critical that people on a LCKD choose desirable low carbohydrate foods that are rich in fiber. In addition, a ketogenic diet should maintain moderate protein intake of around 1.5 g/day per kg of respective body weight [23]. If people consume red meat and organ meats, then they should be able to obtain adequate amounts of iron as well. Additionally, the consumption of small amounts of leafy greens, nuts, berries, and resistant starchy vegetables, all of which are optional ketogenic foods, could potentially maintain healthy gut microbiota [23]. 

Currently, scientists do not have any data on the long-term effects of the ketogenic diet on the gut microbiome. Based on various studies, many predict that the diet will positively affect the microbiome by increasing the *Bacteroidetes* and *Bifidobacteria* species associated with improved health and decreasing microbial species known to increase health risks. In fact, a study found that the disrupted gut microbiota of epileptic infants was improved with a one-week ketogenic diet, which managed to increase their *Bacteroides* amount by ~24% [24]. Another 6-month study on children with refractory epilepsy found a significant decrease in *Firmicutes* and an increase in *Bacteroides* although the overall diversity decreased [25]. 

Studies have shown that a low ratio of *Firmicutes* to *Bacteroidetes* is an indicator of a healthy microbiome [26]. A few studies found that obese patients were more likely to have a higher *Firmicutes* to *Bacteroidetes* ratio [26,27,28] and higher levels of short chain fatty acids (SCFAs) in their stool [5]. However, another study found that obese patients showed an increase in *Bacteroidetes*, while *Firmicutes* remained the same [29]. Therefore, it appears that reducing obesity with the KD may result in positive changes in the microbiome. A study by Basciani et al. [30] recently analyzed the changes in the gut microbiota in obese, insulin-resistant patients who followed isocaloric ketogenic diets which varied in their source of proteins. The very low-calorie ketogenic diets (VLCKDs) contained either whey, vegetable, or animal proteins. The data indicated all groups had a decrease in relative abundance of *Firmicutes* and an increase in *Bacteroidetes* after 45 days. However, the positive changes were less pronounced in the group that consumed animal protein sources.

Recently, a few short-term studies tested the impact of the KD on patient microbiomes. A study by Nagpal et al. [31] analyzed the effect of a modified Mediterranean Ketogenic Diet (MMKD) vs. the American Heart Association Diet (AHAD) on the microbiome of patients with normal cognition or mild cognitive impairment. They found that the MMKD did not show significant changes in the *Firmicutes* or *Bacteroides* phyla at 6 weeks. However, they did see a decrease in the family *Bifidobacteriaceae* and an increase in family *Verrucomicrobiaceae*, which was considered a positive change. Furthermore, the beneficial SCFA, butyrate, increased in the MMKD. The presence of butyrate has been known to increase gut health [31]. 

## 3. The Effect of the Ketogenic Diet on the Epigenome

Epigenetics refers specifically to changes “on top” of the genome that can modify and alter levels of gene expression. These epigenetic markers are heritable, yet recent research suggests that some changes can be reversed or occur through environmental changes [20]. The modifications of the genome involve DNA methylation, changes to chromatin structure, histone modification, and noncoding RNAs. Most notable are histone modifications. For example, the N-terminal of histone tails can be acetylated, methylated, phosphorylated, ubiquitinated, or SUMOylated. Histone deacetylases (HDACs) are enzymes that can remove acetyl groups and condense the chromatin. Similarly, sirtuins (SIRTs) are also capable of deacetylating histones. Histone lysine methylation can either activate or repress a gene’s activity based on the exact location and number of methyl groups added to the histone tail [32]. Research has found that most epigenetic modification occur during early embryogenesis, but the genome can acquire changes later in life. Some of the later epigenetic modifications are caused or modified because of diet [32].

Some ketogenic food sources that positively regulate epigenetic activity are cruciferous vegetables, dietary fiber, foods rich in long-chain fatty acids, and berries, such as raspberries [20]. The benefits of some of these food sources have a multitude of positive effects. For instance, black raspberries not only positively affect methylation patterns in the WNT-signaling pathway, but they also profoundly impact the microbiome make-up (increased *Lactobacillus*, *Bacteroidaceae*, and anti-inflammatory bacterial species), and increased production of butyrate by fermentation in the gut [20]. Thus, it appears that diets rich in certain foods can positively modify genes that increase overall cell health.

The benefits of the ketogenic diet might also go beyond treating existing disease, and instead help prevent chronic and degenerative disease [23]. A literature review by Miller et al. [23] argued that a state of nutritional ketosis will positively affect mitochondrial function and enhance resistance to oxidative stress and noted that the ketones directly up-regulate bioenergetic proteins that influence antioxidant defenses [23]. According to Boison [33], “Ketone bodies, such as β-hydroxybutyrate (BHB), and their derivatives have received the most attention as mediators of the anti-seizure, neuroprotective, and anti-inflammatory effects of KD therapy” [34,35,36]. The ketogenic diet’s mechanism of action might be due to increased levels of adenosine [37,38], which blocks DNA methylation and, thus, exerts an epigenetic change. A study in epileptic rats subjected to the KD therapy found ameliorated DNA methylation mediated changes in gene expression by increasing adenosine [39], which blocks DNA methylation [40]. It is also being studied for its role in the aging process since it is linked to the positive regulation of epigenetic modifications, such as nuclear lamin architecture [41], reduced telomere length [42,43], DNA methylation, and chromatin structure [44]. 

The effect of the ketogenic diet on brain health appears to be well supported and is due specifically to the production of BHB [23]. They found that BHB is more than a fuel molecule; it plays important roles in cell signaling. The signaling functions of BHB link the effects of environmental factors on epigenetic regulation and cellular processes since it is an endogenous class 1 HDAC inhibitor [45]. Thus, a ketogenic diet has been linked to increased global histone acetylation, with a specific increase in the expression of protective genes, such as Foxo3a [46]. 

Evidence also suggests that BHB can have a direct epigenetic effect via a novel histone modification known as β-hydroxybutyrlation of H3K9, which results in improved gene regulation in the hypothalamus and improved overall aging [47]. Furthermore, the energy carrier molecule, nicotinamide adenine dinucleotide (NAD) is important in oxidative respiration. In its oxidative state (NAD+), NAD also acts as a cofactor for sirtuin enzymes and poly-ADP-ribose polymerase (PARP). Sirtuins and PARP play roles in gene expression, DNA damage repair, and fatty acid metabolism [46]. The energy available to a cell is measured by the NAD+/NADH ratio, which is modified by the utilization of glucose versus BHB as a fuel source [48]. During a ketogenic state, more NAD is found in the oxidative state which allows sirtuins and PARP to be more active. Additionally, catabolism of BHB into acetyl-CoA, another energy carrier molecule, raises acetyl-CoA levels. It has been found that the production of two moles of acetyl-CoA using BHB as the precursor reduces only one mole of NAD+ to NADH. However, four moles of NAD+ are produced by glucose metabolism. Thus, the ketogenic diet creates excess NAD+ for the cell and has a positive impact on the redox state of the cell [48]. This might have positive impacts on the activity of NAD+ dependent enzymes, such as sirtuins. Newman et al. [49] found that increased acetyl-CoA favors both enzymatic and nonenzymatic protein acetylation, specifically in the mitochondria, which improves overall mitochondrial function. 

BHB produced by a ketogenic diet may also increase the efficiency of ATP production in the mitochondria and reduce the number of free radicals. As a result of the positive impacts of BHB, one study found that BHB precursor molecules improved cognition and disease progression in an Alzheimer’s mouse model [50]. Additionally, the presence of BHB showed improvement in a case study of a patient with Alzheimer’s disease [51]. The presence of D-β-hydroxybutyrate protect neurons from oxidative damage by reducing the cytosolic NAD+/NADPH ratio, resulting in an increase in the antioxidant agent known as reduced glutathione [52]. BHB also inhibits NF-kB, which is known to regulate the expression of multiple pro-inflammatory genes. This results in a diminished pro-inflammatory response [52]. Similarly, the BHB precursor, 1,3 butanediol, also modulates the expression of the inflammasome via histone β-hydroxybutyrlation. Thus, it reduces the expression of caspase-1, IL-1B, and IL-18 [53], which are inflammation markers. A study in *C. elegans* found that BHB alone could extend their life span [3]. Thus, the endogenous effects of BHB produced by a ketogenic diet might enhance health and increase longevity.

## 4. The Effect of the Ketogenic Diet on Weight Loss

According to recent Harvard models, 50% of the children today are likely to be obese by the age of 35 years [9]. As scientists try to determine the most effective strategies to combat the obesity epidemic, many studies have emerged that compare the health outcomes of different diets. A recent meta-analysis of seven random-controlled trials using diazoxide or octreotide for suppressing insulin secretion in obese patients found that it led to reduced body weight, fat mass, while maintaining lean mass [54]. However, the cost of artificially reducing insulin levels was an increase in blood glucose levels. While these studies seem promising as an indicator of biomarkers that can stimulate weight loss, it seems more logical to help patients achieve lower insulin levels via changes to their diet. The reduction of carbohydrate intake naturally reduces blood glucose levels, thus reducing insulin as a result. Many studies have now demonstrated that the ketogenic diet reduces both blood glucose and insulin levels [55,56,57]

A study conducted by Fumagalli et al. [58] analyzed the genetic profiles of patients and looked at the impacts on metabolism. They specifically looked at human CHC22 clathrin, which plays a central role in intracellular traffic of insulin-responsive glucose transporter 4 (GLUT4). The GLUT4 pathway is the dominant mechanism used by humans to remove glucose from the circulating blood after a meal. They found two major gene variants, one which is more frequent in farming populations than in hunter-gatherers. Hunter-gatherers have the gene that allows GLUT4 to be sequestered more effectively and thus have an inherent increased risk of insulin resistance. It is hypothesized that as humans became farmers and increased glucose in the diet, it was beneficial for the blood sugar to be lowered more easily with the newer form of CHC22. Thus, people with different forms of CHC22 are likely to differ in their ability to clear blood sugar after a meal. The people with the form that allows blood sugar levels to remain elevated could eventually lead to diabetes in the face of a high-carbohydrate load in the diet. This new finding might explain why some patients are successful on a high-carbohydrate low-fat diet, while others prefer to maintain weight with a low-carbohydrate, high-fat diet [58]. 

The importance of dietary adherence is of great concern for the success of any diet study. The study conducted by Shai et al. [59] that was able to control for the feeding of at least one meal a day (cafeteria meal), might better reveal the true effects of a sustained ketogenic diet. The Shai study [59] compared a low-fat, restricted-calorie diet (LFD), a Mediterranean, restricted-calorie diet (MD), and a low-carbohydrate, non-restricted calorie diet (LC) on 322 moderately obese subjects over a period of two years. The dietary adherence was >85% at the end of two years. This study instructed the LC group to be ketogenic for the first 2 months (<20 g/day) and gradually increase to 120 g/day of carbohydrates. The results found that the greatest weight loss occurred in the low-carb group and both the LC and MD were more effective than the LFD. Although, the weight loss during the first 3 months in the LC group was significantly greater than either of the other two groups, as carbohydrates were added back into their diet, their weight rebounded back to a level close to the MD group. Shai et al. [59] found that one of the benefits of the LC group was the similar calorie deficit achieved even though it was not a calorie-restricted diet. The researchers propose that a LC diet may be the optimal choice for individuals that cannot follow a calorie restricted diet since these subjects will be permitted to eat until satiated but will still most likely end up lowering their total caloric intake. 

A similar long-term (56 week) ketogenic study was conducted on 66 obese people with a BMI >30 [60]. All patients were instructed to eat <20 g of carbohydrates in the form of green vegetables and salads for 12 weeks and then they could increase the carbohydrates to 40 g/day for the remainder of the study. The weight and body mass index of all patients decreased significantly. More interestingly, the patients were advised to maintain a state of nutritional ketosis and they were able to show continued decreases in both BW and BMI throughout the study. Consequently, this study did not show the plateau and gradual increases seen in the Shai study [59] which allowed the reintroduction of carbohydrates after the initial weight loss period. A similar study by Samaha et al. [61] also found that patients lost significantly more weight on a 30 g/carbohydrate per day diet for six months compared to a LFD. Another possible benefit from the ketogenic diet is that there is a measurable biomarker that signifies dietary adherence, which is β-hydroxybutyrate (BHB). When an individual is in ketosis, the body will begin ketone production and the level of BHB in the blood will be over 0.5 mmol. Studies that include this measurement can therefore confirm dietary adherence and determine the true effects of the diet on health outcomes, like weight loss. Mohorko et al. [57] conducted a 12-week ketogenic diet study on obese patients who were calorie restricted (1200–1500 kcal) for the first two weeks and then were instructed to eat ad-libitum for hunger for the remaining weeks while eating the macronutrient composition necessary to remain in a state of nutritional ketosis. BHB was measured throughout the study and patients maintained levels above 0.5 mmol throughout the 12 weeks. Patients showed significant weight loss in both the men and women groups (average of (-)18 kg for men and (-)11 kg for women). Interestingly, as the diet progressed, the patients Fat Mass (FM) became the largest component of weight loss and it significantly correlated with BHB. Another valuable outcome in this study was the reduction of the hunger hormone, leptin, as well as a slight increase in energy expenditure, even while weight decreased throughout all 12 weeks. Another long-term study was done by Hallberg et al. [2] which followed diabetic patients on a ketogenic diet for one year. At the beginning of this study, 92% of the patients in the ketogenic group were obese. These patients were instructed to eat less than 30 g of total carbohydrates per day and the goal was to maintain BHB blood levels of 0.5–3.0 mmol/L. These patients had an average of 12% decrease in body weight, with some patients achieving as high as ~40% change. The patients who were in the standard care diet group (American Diabetic Association recommended diet) did not see any significant change in body weight [2]. 

A short-term, 4-week ketogenic diet (KD) on 20 obese Chinese females had profound outcomes [62]. In this study, compliance to the diet was measured with urinary ketone strips. These participants were given a monitored 4-week normal diet which was followed up with a 4-week KD with the same daily caloric intake but a drastic reduction in carbohydrates to <10% of calories. The effect was a significant decrease in body weight, body mass index, waist circumference, hip circumference, body fat %, and decreased fasting leptin levels. Similar positive outcomes were seen in other KD diet studies [56,63,64]. Similarly, a recent meta-analysis concluded that very low-calorie ketogenic diets are a very effective strategy for treating obesity [65]. An 8-week study conducted by Goss et al. [66] compared the very low carbohydrate diet (VLCD) (<10% carbohydrates) to a low-fat diet in older obese adults with BMI between 30 and 40. This study precisely measured fat loss with DXA and MRI measurements. Both groups exhibited decrease in total fat, but the VLCD experienced ~3 fold greater decrease in visceral adipose tissue and a significant decrease in intermuscular adipose tissue with a 5-fold greater reduction in total body fat mass.

Another long-term study monitored weight loss as well as changes in visceral fat mass using DEXA. The study by Moreno et al. [67] compared a very low-calorie ketogenic diet (VLCK) to a low-calorie (LC) diet as a treatment for obesity over two years. Participants in the active stage consumed 600–800 kcal/day and <50 g of carbohydrates per day until they were 80% of target weight loss goals (stage 1). Urinary ketone strips were used during stage 1 to confirm a state of ketosis. Then they used a standard low-calorie diet (10% below total metabolic expenditure) during stage 2 until they achieved another 20% weight loss, followed by long-term maintenance of weight loss in stage 3. The comparison control group used the low-calorie diet throughout the study to achieve weight loss. The weight loss in kilograms in the VLCK diet was double that of the LC diet throughout most of the study and remained significant. The amount of visceral fat loss in the VLCK diet group was 3X greater than the control group while preserving lean body and skeletal bone mass. The main side effects recorded in the VLCK were fatigue, headache, constipation, and nausea. However, none of these side effects were severe enough to cause the patients to drop out of the study and most subsided within the first month [67]. 

A meta-analysis conducted by Bueno et al. [68] compared randomized controlled trials of very low carb ketogenic diets (VLCKD) with low fat diets for 1 year. This study found a significant difference in decreased body weight for the VLCKD group. Another study compared a KD (<30 g carbohydrates/day) with two control groups (standard American diet (SAD) without exercise and SAD with 3-5 days of exercise for 30 minutes) over ten weeks [69]. The KD outperformed the other control groups in all variables tested, with 5 out of 7 being statistically significant. The patients showed significant decreases in body mass index (BMI), body fat mass (BFM), and weight while their resting metabolic rate (RMR) increased. The RMR in the experimental group produced a positive, sizeable change with a magnitude of slope that was more than 10X the two control SAD groups. These results reveal that diet plays a more significant role in outcomes than exercise [69].

The ability to control hunger is also a key component to weight loss success. Castro et al. [70] evaluated patients from the very low-calorie ketogenic diet (VLCK) study and found a negative correlation between BHB levels and the urge to eat and feelings of hunger during the phase of maximum ketosis, even though there was no significant change in ghrelin hormone. This result is supported by other large investigations in overweight and obese adults which also found that low-carbohydrate diets were more effective in controlling hunger than low-fat diets [71,72]. A 2-week study conducted by Choi et al. [73] compared varying nutrition drinks on weight loss in obese adults. There were three groups: 4:1 fat to protein and carbohydrate ratio, 1.7:1 ratio with increased protein, and a balanced nutrition drink with similar carbohydrates to recommended dietary advice. All groups decreased body weight and body fat mass, but only the 1.7:1 KD-group maintained protein mass. Furthermore, only the KD groups improved blood lipid levels with appetite reduction. Since this was a nutritional drink feeding study, all the groups had similar caloric reduction; thus, results were due to macronutrient composition. In addition, levels of ketosis were strongly related to positive differences in food cravings, alcohol cravings, physical activity, sleep patterns, and sexual activity [73]. This outcome might also be supported by a recent finding that postprandial glycemic dips were the best predictor of appetite and energy intake following a meal and large glycemic dips are usually associated with high carbohydrate consumption [74]. Furthermore, a study showed that high carbohydrate meals had a greater impact on brain reward and homeostatic activity in ways that could impede weight loss maintenance [75]. Interestingly, the increased brain activity findings were partially associated with higher insulin levels, too. Thus, the ability of the KD to reduce hunger, lower glycemic fluctuations, and reduce influences on areas of the brain associated with addiction are all positive signs that a ketogenic diet should be considered as a treatment option for obesity.

One of the major concerns for rapid weight loss is the lowering of the resting metabolic rate (RMR). This bodily change can lead to weight regain, which is known as adaptive thermogenesis. Thus, it is typical for hunger to increase and energy expenditure to decrease during weight loss, which is a hindrance to long-term weight loss maintenance. Gomez-Arbelaez et al. [76] tested this outcome in subjects on the very low-calorie ketogenic (VLCK) diet study and followed them for 2 years. In this study, twenty obese patients lost 20.2 kg of body weight after four months and sustained this weight loss without the expected reduction in RMR. Authors of the study hypothesize that RMR did not drop because the subjects maintained their lean body mass. DEXA scans revealed that although they lost ~20 kg of fat mass, they only lost 1 kg of muscle mass. This conclusion was also supported by normal renal activity and positive nitrogen balance while subjects maintained their fat loss upon follow-up [76]. 

A study by Hall et al. [77] hypothesized that the development of obesity is “a consequence of the insulin-driven shift in fat partitioning toward storage and away from oxidation resulting from an increased proportion of dietary carbohydrates.” To test this hypothesis, they tested seventeen obese men in metabolic wards with a four-week high-carbohydrate diet followed by a four week, isocaloric ketogenic diet. The results showed that a state of ketosis increased energy expenditure (~100 kcal/d), most likely due to beta oxidation and the partitioning of fuel towards ATP production rather than fat storage [77]. However, this level of energy expenditure change due to a ketogenic diet is not as high as measured in another study. In the study by Ebbeling et al. [78], it was noted that short-term feeding studies do not consider the body’s process of fat adaptation, which takes at least 2–3 weeks, if not longer. Thus, the Framingham study by Ebbeling et al. [78] conducted a randomized trial on 164 patients where they lost weight and were then placed on varying diets of carbohydrate content for twenty weeks to measure changes in energy expenditure. The difference in total energy expenditure was 209–278 kcal/d or around 60 kcal/d increase for every 10% decrease in the carbohydrate percentage of total energy intake. This study concluded that dietary quality could affect energy expenditure independently of body weight. In accordance, Mobbs et al. [79] has suggested that ketogenic diets “reverse obesity by preventing the inhibitory effects of lipids on glycolysis, thus maintaining relatively elevated post-prandial thermogenesis.” Further studies will need to be conducted to evaluate and confirm the exact mechanisms of action. 

More recent studies on the KD are analyzing the outcomes of the diet in conjunction with other comorbidities related to obesity. A small study was conducted by Carmen et al. [80] that followed three obese participants on a 10% carbohydrate KD for 6–7 months that exhibitied comorbid binge eating and food addiction symptoms. No adverse effects were found, and participants had reductions in binge eating episodes and food addiction symptoms. All three lost 10–24% BW and maintained treatment outcomes 9–17 months after initiating the diet and continued adherence to the diet [80]. Another study looked at the outcomes for male and female severely obese patients who also suffered from non-alcoholic fatty liver syndrome (NAFLD) [81]. They used a very low-calorie ketogenic diet of <50 g of carbohydrates and <800 kcal/day. Both males and females showed significant losses in body weight. However, males lost significantly more weight and had greater reductions in waist circumference. The patients also improved their biomarker for NAFLD, which was a reduction in gamma-glutamyl transferase [81]. To determine if the ketogenic diet negatively affects kidney function, Bruci et al. [82] conducted a 3-month very low-calorie ketogenic diet (VLCKD) study for weight loss in obese patients with and without mild kidney failure. All patients were advised to consume <20 g carbohydrates and 500–800 calories per day. The average mean weight loss from initial weight was nearly 20%, participants had significant reduction in fat mass, and 27.7% of the patients with mild kidney failure acquired normalized glomerular filtrate rate. It was, therefore, concluded that a KD not only leads to weight loss but also improvement in kidney function.

Please refer to Table S1 in the Supplementary Materials for a comparison of studies evaluating the KD in relation to weight loss outcomes.

## 5. The Effect of the Ketogenic Diet on Diabetes

According to the latest CDC report, an estimated 30 million people have diabetes and around 84 million have pre-diabetes. That statistic predicts that ~45% of Americans are either diabetic or pre-diabetic. Diabetes is a major health concern that is accompanied by a long list of secondary complications and diabetics are at increased risk of microvascular pathology of the retina, renal glomerulus, peripheral neuropathy, and atherosclerotic disease affecting arteries [83]. Many of these diabetic complications have been linked to elevated levels of glucose over long periods of time, which is measured as hemoglobin A1c (HbA1c) [83]. 

Type 2 diabetes is caused by hyperinsulinemia and insulin levels are directly affected by carbohydrate consumption. Protein intake can cause slight increases in blood glucose and subsequent insulin secretion, but fat consumption has no major effect on either [84]. If hyperinsulinemia is directly affected by nutrient intake, then it could be argued that these blood markers could be controlled by the conscious control of food choices. Of further note, the American Diabetes Association (ADA) recommends a goal of an HbA1c less than 7%, and the American College of Endocrinology sets a target level of 6.5%, even though few patients ever obtain that goal. Thus, Brownlee et al. [83] argued that patients should increase efforts to minimize glycemic variability since it can reduce risks of diabetic complications, independent of HbA1c. The DiRECT study by Lean et al. [85] found that weight loss alone could result in almost 46% of patients achieving diabetes remission at 12 months. Yet, this does not address the issue of diabetic patients who are not overweight. Thus, many scientists are now examining the potential benefits related to diabetes and improved blood markers that can result from eating a ketogenic diet. Although no professional organization in endocrinology or diabetology has focused on the rational use of ketogenic diet for either diabetes or obesity conditions, Kalra et al. [86] argues nutrition should be considered as an integral part of metabolic management of diabetes, and the ketogenic diet should at least be offered as a treatment option. 

Interestingly, the use of a diet low in carbohydrates for the treatment of diabetes is not a new or novel idea. In fact, prior to the invention of insulin, diet was the main intervention used by diabetic patients. The physicians, Dr. Elliot Joslin and Dr. Frederick Allen, were both recommending their patients in the 1920s to eat foods without carbohydrate content, and it highly resembled the current ketogenic recommendations [87]. According to Feinman et al. [88] the number one goal of both type 1 and type 2 diabetics should be glycemic control. It is argued that carbohydrate restriction can benefit diabetic patient blood markers even in the absence of weight loss [88]. This is important since many diabetics are not overweight yet still need to manage their blood glucose levels. The benefits of carbohydrate restriction in type 1 diabetics reduces the error in determining insulin amount to match the increased blood glucose since dramatic spikes are less likely [88]. 

A recent study compared the use of a low-calorie (LC) diet vs. a very low-carbohydrate ketogenic diet (VLCKD) on health outcomes for type 2 diabetics. The VLCKD group approached normal blood sugar level in just 24 weeks unlike the LC group [87]. The VLCKD group reduced insulin doses by half, on average, and sulfonylurea doses were halved or discontinued. The HbA1c levels dropped significantly in the VLCKD to 6.2% vs. 7.5% in the LC group. Thus, the VLCKD group managed to reach both the ADA and American College of Endocrinology target level for HbA1c. According to Hussain et al. [87] the VLCKD was not found to have an adverse effect on glucose metabolism, insulin resistance, or cause chronic dehydration. However, they did caution that diabetic patients should only attempt this nutritional therapy while being closely monitored by a physician to reduce the risk of hypoglycemia since drugs will need to be quickly reduced to match changes in blood markers elicited by the diet [87]. A study by Webster et al. [89] found that type 2 diabetic patients who self-selected to follow a KD reduced their mean HbA1c from 7.5% to 5.9% at the 15-month follow-up. As a result, their HbA1c levels reached the normal range (which is under 6.0%), and they had achieved partial or full type 2 diabetes remission.

A study conducted by Westman et al. [8] compared the effects of a low-carbohydrate ketogenic diet (LCKD) versus a low-glycemic index diet (LGID) on glycemic control in type 2 diabetic patients which was measured by hemoglobin A1C (HbA1c). They enrolled forty nine patients and randomly assigned them to the different diets. Both groups followed group meetings, nutritional advice, and an exercise recommendation. Both interventions showed improvements in hemoglobin A1c, fasting glucose, fasting insulin, and weight loss. However, the LCKD had greater improvements, including a reduction or elimination of diabetes medications in 95% of patients vs. 62% in the LGID group [8]. As mentioned previously, the study by Dashti et al. [60] compared the health outcomes of a ketogenic diet on obese diabetics with high blood glucose levels to non-diabetic obese patients over 56 weeks. This study concluded that all markers, such as body weight, body mass index, blood glucose, total cholesterol, LDL, triglycerides, and urea all showed a significant decrease in both groups throughout the study, with more positive outcomes seen in the diabetic group [60]. The kidney tests also showed normal function. This study demonstrated that the diet is safe to use for longer periods of time in obese diabetic subjects.

A year-long randomized study compared the effects of a very low-carbohydrate ketogenic diet (LCK) versus a moderate-carbohydrate, calorie-restricted, low-fat diet (MCCR) in pre-diabetic or type 2 diabetic patients [90]. The results showed that the LCK exhibited greater improvements in their HbA1c, weight loss, and medication use than those assigned to the MCCR diet [90]. Another randomized controlled study by the same researcher compared the LCK against the diet program based on the online American Diabetes Associations’ “Create Your Plate” diet. The purpose of this study was twofold. The researcher had already seen the benefits of the LCK in a previous study that had personalized intervention. They wanted to see if an online program could be just as successful at helping overweight individuals with type 2 diabetes. The results indicated that the online ketogenic program was more successful in helping patients manage their diabetes by reducing their HbA1c, lowering triglycerides, increasing weight loss, and retention rates were higher than in the control group [91]. In addition, a previous study has discovered that a carbohydrate restricted diet was more successful than a low-fat diet in improving diabetic markers for metabolic syndrome in forty subjects with atherogenic dyslipidemia [92].

A recent study recently conducted at Indiana University was one of the first long-term studies that required use of routine blood tests to determine the patients’ state of nutritional ketosis while maintaining a KD diet. Patients were highly compliant, and experienced improved diabetic conditions [2]. The diet intervention also reversed the diabetic status of some patients, whose HbA1cs became normal. The 2-year follow-up to this study revealed that 74% of KD group remained enrolled [93]. This group had a significant improvement in HbA1c, fasting glucose, and fasting insulin while the usual care group had no changes from baseline. The mean dose of prescribed insulin decreased by 81% and the diabetes reversal increased to 53.5%. Diabetes remission was 17.6% and diabetes complete remission was 6.7% [93]. The long-term success in diabetes treatment for this digitally monitored continuous care intervention group is evidence of the feasibility and adherence of the KD in type 2 diabetes treatment [2,93]. 

Additionally, the study by Shai et al. [59] showed that patients were able to reduce their fasting blood glucose on a low carbohydrate or a Mediterranean diet, while the low-fat group saw the opposite effect. The patients in the low carbohydrate group were also able to significantly decrease their HbA1c [59]. Another meta-analysis that compared very low-carbohydrate ketogenic diets (VLCKDs) to low-fat diets (LFDs) found that the VLCKD showed greater improvements in fasting glucose, insulin analysis, HbA1c, and C-reactive protein [68]. Additionally, a recent meta-analysis of low-carbohydrate or very-low carbohydrate diets found that patients adhering to the diet for 6 months can have diabetes remission without severe complications [94]. Several recent studies on the KD show positive improvements in glycemic profiles [56,66,82,89,95]. 

Currently, the ADA recommends that type 1 diabetics eat a low-fat diet rich in whole grain carbohydrates. One study showed the low-fat diet has not been found to improve HbA1c in all patients, regardless of diabetes state [96]. It looked at the HbA1c outcomes for type 1 diabetics (T1D) who were advised to reduce carbohydrate intake (<75 g of carbs/day) to reduce the need for insulin. The patients in this study had a 50% adherence rate, and those who strictly adhered to the diet reduced their HbA1c by 1.8%. Another randomized trial [97] determined the feasibility of a LC diet (<75 g/day) versus standard carb counting in adults with T1D. Of the ten people in the 12-week study, the LC group exhibited significant decreases in HbA1c, decreased daily insulin use, and reduction in body weight. All of the outcomes in the carb counting group were unchanged. Thus, these T1D patients had positive outcomes without meeting the KD threshold of <50 g/day while consuming significantly less carbohydrates than the typical diet.

Interestingly, some type 1 diabetes patients have taken it upon themselves to treat and control their diabetes with the very low–carbohydrate diet (VLCD), against the advice of current medical professionals. Lennerz et al. [98] evaluated the results of this choice by recruiting type 1 diabetics who self-selected to follow a VLCD (<30 g/day). They found these patients on a social media site and then asked for permission to contact physicians and confirm health outcomes. Shockingly, 97% of the patients were able to achieve the ADA glycemic targets for HbA1c with an average of 5.6% and a mean daily insulin dosage of 0.40 U/kg per day. Participants in this group reported increased levels of overall health, increased satisfaction with diabetes management, and decreased number of adverse events. These results are unprecedented in type 1 diabetic patients. If these outcomes are confirmed in clinical trials, the chronic health issues associated with type 1 diabetes could be prevented or significantly reduced by diet alone. Almost one-fourth of these patients did not discuss their VLCD with their care providers, which means they were making these changes without the support of their physicians. Even in an intensively treated group in the Diabetes Control and Complication Trial, the best HbA1c achieved was 7.2%, but that was coupled with increased rates of hypoglycemia [98]. 

Although there are only a few randomized controlled trials evaluating the effects of the KD on diabetes, there are some recent case studies and qualitative studies that shed some light on the issue [55,64,99,100]. The positive outcomes in these studies might reflect the motivation of these patients who opted or volunteered to ensue KD diets. A paper by Walton et al. [64] presented 11 case studies on women with T2D that volunteered to eat a KD with <30 g of carbohydrate per day. Their HbA1c was > 6.5% and dropped to 5.6% with diabetes reversal. Another case study by Lichtash et al. [99] involved a women patient with T2D and normal weight. After failed glycemic control with standard care, she voluntarily began a KD with intermittent fasting. Her HbA1c dropped from 9.3% to 5.8% after 14 months while maintaining her weight. Similarly, Wong et al. [100] examined type 1 and type 2 diabetics who opted to do a KD for >3 months. Participants reported better glycemic control, decreased medicine use, weight loss, and satiety. Most of these patients expressed the KD as a normal way of eating and plan to continue for the rest of their lives. A similar T2D cohort was recruited [55] for a retrospective study on 49 patients who followed KD for > 3 months and compared their outcomes to 75 patients who followed usual care (UC). 100% of the KD cohort either discontinued or reduced insulin dosage while only 23% of UC did. The KD cohort had a greater reduction in fasting plasma glucose, weight loss, as well as a superior reduction in HbA1c compared to UC. Thus, it seems that those patients who opt to follow the diet are having positive outcomes.

Please refer to Table S2 in the Supplementary Materials for a comparison of studies evaluating the KD in relation to diabetic outcomes.

## 6. The Effect of the Ketogenic Diet on Lipidology and Cardiovascular Risk

Cardiovascular disease (CVD) and its risk factors are a major health issue in industrialized nations. Moreover, large epidemiological studies are starting to show that CVD is becoming a larger problem in developing or low-income countries as well [101]. There has been a long-standing viewpoint that a diet high in saturated fat is unhealthy and will eventually lead to cardiovascular disease. Many hypothesized that a diet rich in saturated fat will increase LDL, and thus more fat in the blood leads to fat deposits in the vessels, resulting in increased risk of cardiovascular disease [102]. This idea was fortified by Ancel Keys in his 7-country study and eventually led to the diet-heart hypothesis [102]. Moreover, the United States accepted the idea proposed by Keys and adopted the low-fat diet (LFD) as the optimal diet to fight the increasing levels of CVD in the U.S. Additionally, the mainstream view for decades was that high total cholesterol also leads to atherosclerosis and cardiovascular disease [103]. As a result, the prescription of the LFD that consisted of ~60% energy from carbohydrates became the standard of care for physicians starting in the 1980s [98]. According to the 2015 Dietary Guidelines for Americans, people are still recommended to consume a diet that limits saturated fat intake to less than 10%, with some organizations placing even stricter limitations and advising around 7% [104]. However, randomized controlled trials have started to question the validity that saturated fat intake and a single blood marker, LDL, can accurately predict risk. Many scientists now argue for the need to analyze specifically how different types of macronutrients that replace saturated fat in the diet are impacting risk [105]. It is also important to consider the data regarding LDL as the single biomarker chosen to monitor and determine cardiovascular risk [105]. 

New research is also starting to question that mindset set forth by Ancel Keys, and many scientists have argued that the global dietary recommendations should be revisited and updated [106]. For example, a recent analysis of the literature done by Ravnskov et al. [103] compiled all the data on PubMed from initial to 2015 on over 68,000 patients. Ravnskov et al. [103] argued that that if the main goal of prevention of disease is prolonging life, then all-cause mortality should be the measurement used for determining health outcomes. Interestingly, they found that 30% of patients showed no association between LDL and all-cause mortality, while 70% showed a statistically significant inverse relationship. Contradicting the diet-heart hypothesis, they also found that the 4-year mortality among patients with the highest levels of LDL were almost 36% lower than those patients with the lowest LDL levels. Furthermore, the patients placed on statins had higher rates of mortality risk than those with the highest LDL. The results of these studies question the standardized method of using total cholesterol and LDL as the biomarkers of coronary heart disease. 

Thus, if total cholesterol and LDL are not true indicators of cardiovascular risk, then one must ask what other blood markers could serve as better indicators of coronary heart disease. In a review by Feinman et al. [88] they argue that the best indicators of CVD risk are ApoB [107], the ratio of TC/HDL, increased levels of small dense LDL particles (sdLDL) [108,109], and the ratio of ApoB to ApoA1 [88]. If these markers are, in fact, a better indicator of disease risk, then understanding the effect of diet on these other biomarkers is of great importance. One study by Krauss et al. [110] compared patients who consumed diets of varying carbohydrate intake (54%, 39% or 26%) with the amount of saturated fat varying between 7% or 15%. This study showed that a high saturated fat intake, combined with carbohydrate restriction (26%) did raise total LDL. However, the higher total LDL levels were due to an increase in the larger sized LDL particles, which are less atherogenic than the sdLDL, and the patients saw a subsequent lowering of the sdLDL particles [9,110]. 

A large prospective study called the European and Prospective Investigation into Cancer and Nutrition Study (EPIC) also found that diets high in glycemic load (GL) and glycemic index (GI) were associated with a greater risk in Cardiovascular Heart Disease (CHD) [111]. Glycemic index is a measurement of the ability of carbohydrates to increase blood glucose levels. The glycemic load is the product of the GI of a particular food and its available carbohydrate. This study included around 520,000 men and women between the ages of 35 and 70 over a period of 8 years [111]. The study found a greater risk of CHD with higher sugar consumption. Their findings supported other observational studies that suggest that replacing saturated fat with sugar or refined carbohydrates might increase cardiovascular risk, rather than lower it [112,113]. Additionally, the very large PURE study recently showed that a diet higher in saturated fat did increase LDL, but also increased HDL, lowered triglycerides (TG), lowered the TC/HDL ratio, and lowered the ApoB/ApoA1 ratio [106]. They also found that the diets high in carbohydrate intake had the complete opposite effect on these atherogenic biomarkers. The benefit of the PURE study is that it revealed the risk associated with varying macronutrient composition in diets from over 5 continents in 18 countries, regardless of cultural food trends. Thus, it was a global look at the effect of dietary patterns on health regardless of background and ethnicity. The PURE study concluded their findings do not support the current recommendations to limit total fat intake to 30% of energy and saturated fat to less than 10%, and the recommended amount of <7% saturated fat might even be harmful. Instead, they argue that individuals who eat a diet high in carbohydrates might benefit by replacing some of those carbs with fat [106]. According to the PURE study, the ApoB to ApoA1 ratio was the strongest lipid predictor of myocardial infarction and ischemic stroke. Since this biomarker has been found to increase with carbohydrate intake, they concluded that this factor could provide the mechanistic explanation for higher risks seen in people with the highest carbohydrate intake [106]. This idea was supported by a recent article on Medscape, which argued that the predictive power of the ApoB to ApoA1 ratio was superior to other biomarkers to assess CV risk [114]. It also mentioned adding other lipid parameters to the ApoB/ApoA1 ratio did not improve the predictive power.

A study done by Lu et al. [115] compared the ability of either the ApoB/ApoA1 ratio or LDL to predict coronary heart disease (CHD) in normal and overweight patients. They found every quartile increase in the ApoB/ApoA1 ratio showed an increase in CHD prevalence. Meanwhile, the increases in LDL quartiles did not predict the highest percentages of CHD [115]. The ratio had an even stronger predictive capability in the overweight subjects. Furthermore, other studies have also supported the findings of the PURE study. One study conducted on postmenopausal women found an inverse relationship between dietary saturated fat intake and atherogenic disease progression [116]. Another study previously mentioned even found a positive association between plasma phospholipids and CHD mortality [117]. According to another study conducted by Dreon et al. [108], a decrease in saturated fat intake did lower total LDL, but it appeared to only reduce the amount of the large, buoyant LDL particles. They argue that more emphasis on CVD risk should be placed on high levels of triglycerides (TG), decreased concentration of HDL, and increased amounts of sdLDL particles. If these biomarkers are potentially more effective predictors of coronary heart disease, then the analysis of a diet’s effect on these lipid markers is of great importance [109].

Only a few studies have looked at the health impact of very high fat consumption (VLCKD) on overall health (which could include analysis of weight maintenance, lipid profiles, and inflammation markers [69]. To accurately determine the effect of a KD on cardiovascular risk markers, it is important to only look at studies that restricted carbohydrates below 50 g/day to ensure the patients would be in a state of nutritional ketosis. One study compared a KD to the standard American diet (SAD) and the SAD plus exercise. Not only did the KD outperform the other groups in multiple health outcomes, but it also showed a much more significant decline in triglycerides [69]. Another study compared a LC diet group (<30 g/day) to a LF diet in obese patients after 6 months [61]. Once again, the LC group had a drastic decrease in TG, while no significant difference was seen in total cholesterol (TC), HDL or LDL. This led investigators to conclude that the LC diet did not have adverse effects on serum lipid levels. 

The impact of the prescribed low-fat diet versus diets higher in fat on cardiovascular lipids levels are beginning to emerge. One 2-year diet study compared the effect of a low-fat diet (LFD), low carbohydrate diet (LC), and a Mediterranean diet (MD) on lipid profiles of overweight patients [59]. The LC group had a significant decrease in triglycerides and the total cholesterol/HDL ratio decreased the most in the LC group. Their ratio decreased by 20% compared to a 12% decrease in the LF group [59]. The beneficial biomarker, HDL, increased in all groups, while the LDL changes were similar in all groups, which has also been noted in other studies [118]. A metabolic ward study of shorter duration conducted by Hall et al. [77] also found that triglycerides decreased in the reduced carbohydrate group. However, they saw the LDL levels increase in the LC group. Meanwhile, the Choi et al. [73] study mentioned earlier did not find an increase in LDL. It was conducted on obese patients with tightly controlled nutrition drinks, which had similar calorie reduction. Only the KD groups improved blood lipid profiles while reducing appetite. The KD groups saw a decrease in triglycerides and LDL, and no significant change in HDL [73]. 

Another 6-month study compared a low-calorie KD to a low-calorie diet in obese patients; some were diabetic. They found that both the diabetic and non-diabetic patients in the KD group showed the best lipid outcomes [87]. They found a significant decrease in triglycerides, a decrease in total cholesterol, a decrease in LDL, and an increase in HDL. A study conducted by Walton et al. [64] followed 11 women with type 2 diabetes for 90 days on a KD. The women in this study had increased HDL, a significant decrease in TG, and a significant decrease in the TG: HDL ratio, although LDL levels were not significantly changed. Another cardiovascular benefit was the lowering of the patient’s systolic and diastolic blood pressure. When evaluating type 1 diabetic patients who self-selected to be on a LCD, they found that these patients showed a decrease in TG, while having increases in HDL, TC, and LDL [98]. The researchers hypothesized that the total LDL elevation on the KD, if associated with a low TG, may reflect an increase in the large, buoyant lipoprotein particles which are considered a lower risk subtype. When the KD was followed for one year in type 2 diabetics and adherence was confirmed with BHB, it was noted that TG decreased by 24%, HDL increased 18%, LDL increased 10%, while ApoB was unchanged [2]. Although these lipid changes are considered favorable, the increase in LDL seen in some groups is still an area of concern. One analysis suggested that the risk from a slight increase in LDL might be offset by emphasizing the consumption of unsaturated fatty acids rather than saturated fatty acids [9]. 

The DIETFITS study also concluded that the increase in saturated fat intake may improve overall lipid profiles if they are adhering to a high-quality, whole-food based, low carbohydrate diet [104]. One major area of concern would be whether the KD would have these same beneficial changes in patients with dyslipidemia. A 56-week study tested the effect of the KD on obese patients with and without high cholesterol levels [119]. It is important to note that these patients were instructed to include 5 tablespoons of olive oil into the diet, which is a form of unsaturated fatty acids. Throughout the experiment, the patients saw continuous improvements in their lipid markers. Not only did both groups have decreased LDL, decreased TG, and increased HDL levels, but the patients with high cholesterol levels also ended the study with blood profiles that were more like normal subjects. 

A more recently published case study on a young man who used a Mediterranean KD diet for treating his IBS had some interesting findings [120]. The doctors looked at more detailed lipid subfractions to determine the lipid outcomes of cardiovascular risk, which was unique. First, the authors mention that a typical lipid profile analysis would suggest the diet was having adverse effects on the patient. His total cholesterol changed from 160 to 450 mg/dL, even though a portion of that was due to increased HDL levels. Many argue that HDL-P is a superior predictive measure of good cardiovascular health. The HLD-P in this patient increased from 5699 nmol/L to 12,080 nmol/L. The current association between LDL-C and cardiovascular risk is driven by atherogenic small dense and/or oxidized LDL. It is believed that these two components can penetrate the endothelium of blood vessels and contribute to plaque formation [121,122]. Yet, large LDL are not associated with cardiovascular risk and may provide a protective effect. This patient saw an increase of LDL from 90 to 321 mg/dL. The LDL subfraction revealed that almost the entire increase in his LDL-C was caused by an increase in large LDL, while his small and medium LDL decreased by almost 10%. Thus, these authors argued that the typical analysis of lipid profiles from patients on ketogenic diets may not accurately reveal risk unless more detailed lipid subfraction tests are conducted. 

Please refer to Table S3 in the Supplementary Materials for a comparison of studies evaluating the KD in relation to lipidology outcomes.

## 7. The Effect of the Ketogenic Diet on Cancer

Cancer currently remains the second leading cause of death (~22%) in the United States and is second only to heart disease [123]. Typically, cancer occurs in adults because of multiple mutations in numerous genes, genes that usually regulate cell growth and proliferation [124,125,126]. Today it is the accepted model that as many as six mutations need to occur to produce cancer (usually to oncogenes and tumor suppressor genes). Oncogenes are genes that regulate cellular pathways that can increase cellular growth, while tumor suppressor genes regulate pathways that inhibit abnormal cell growth. As the mutated cell population expands, it accrues the necessary changes to ignore growth control signals, avoid apoptosis, escape immune surveillance, and creates an environment to thrive (using mechanisms like angiogenesis and tolerance for anoxic environments) and eventually the capability to metastasize [124]. These mutations can result from many causes, such as DNA replication errors, failed DNA repair mechanisms, mutagen exposures, or increased reactive oxygen species [127]. 

Consequently, preventative mechanisms that could lower cancer incidence would be related to reducing these external causes or activating internal pathways to reduce cellular error. Additionally, epidemiologic evidence linking obesity to elevated cancer incidence found that 14% and 20% of all cancer deaths in men and women, respectively, are due to being overweight and obese [128]. As a result, the Annual Report to the Nation on Cancer emphasized the increasing contribution of obesity on cancer incidence [129]. One mechanism believed to contribute to obesity’s role in cancer is the increase in adipocytes in the body, which can increase circulating levels of insulin and Insulin Growth Factor 1 (IGF1) hormones. These hormones bind receptors in many cell types and activate P13K/AKT signaling pathways that increase cell survival and upregulate transcription factors that promote cell proliferation [130]. Both hormones also increase glucose uptake into cells, resulting in increased energy molecules being available for cell growth. Insulin is an anabolic hormone that promotes glucose uptake into cells, reduces the release of fatty acids from adipocytes, prevents ketone production in the liver, and stimulates fat and glycogen storage [131]. Additionally, many recent publications support the idea that prolonged, increased levels of serum insulin is likely to promote cancer growth [132,133,134].

The alterations in the metabolism of cancer cells were first described by Warburg et al. in 1927 [135] It was discovered that cancer cells acquire mutations in critical genes that change the way cancer cells acquire energy. First, cancer cells use glycolysis for ATP production and reduce their dependency on the oxidative cellular respiration in the mitochondria. This results in the cancer cells gaining only 2 ATP per glucose molecule instead of the average 36 ATP from typical cellular respiration processes, resulting in an enormous demand for glucose. Secondly, it allows the cancers cells to rapidly divide even in the absence of oxygen, since glycolysis is an anaerobic process that occurs in the cytosol. Currently, altered metabolism has been described as a primary signature of cancer [125,136,137]. Since this discovery, the use of metabolic therapies for dealing with cancer have been overshadowed by discoveries in the genetics and molecular signatures of cancer [138]. 

Therefore, it seems reasonable to hypothesize that diet could have profound effects on reducing cancer risk, especially if that diet is known to decrease body weight, lower insulin levels, and target the metabolic weaknesses of cancer cells. Some researchers hypothesize that the ketogenic diet might reduce cancer risk because it capitalizes on the reduced expression of ketolytic enzymes in cancer cells [48]. The diet would starve the cancer cells by reducing their ability to utilize glucose, while normal cells can adapt and begin utilizing ketone bodies for their energy demands. Another potential benefit could be the decrease in insulin that results from being in nutritional ketosis, which would reduce insulin-like growth factors that support cancer proliferation [48]. Especially given the fact that 20% of all cancer cases in North America can be attributed to obesity and 38% of all attributable cancer cases are linked to the increase in BMI since 1982 [139]. There have also been numerous studies that have linked cancer risk to hyperinsulinemia [140,141,142,143,144]. It is suggested that insulin resistance leads to hyperinsulinemia, and insulin has both pro-mitotic and antiapoptotic activity that may assist in tumor progression. Thus, any diet that can reduce obesity and lower insulin levels, such as the ketogenic diet, might reduce cancer risk. 

Support for a KD as a mono-therapeutic approach for treating cancer is demonstrated in many mouse models. However, due to the heterogeneity of these studies (types of cancers, KD protocol, length of study, etc.), we discuss them separately. Poff et al. [145] tested a KD on systematic metastatic cancer in mice. They found that KD alone significantly decreased blood glucose levels, reduced tumor growth, and improved mean survival time by 56.7%. A similar study looked at the effect of the KD on mice with gastric tumor cells. Both tumor growth and mean survival time were improved [146]. In one study, Allen et al. [147] found that a KD reduced tumor growth in lung cancer xenografts. 

In another study, they tested the use of a calorie-restricted KD on the growth and vascularity of malignant mouse astrocytoma (CT-2A) and human malignant glioma (U87-MG). When compared to an unrestricted high carbohydrate standard diet, they found that tumor growth decreased by 65% for CT-2A and 35% for U87-MG tumors [148]. They also found that signs of angiogenesis were reduced in the calorie restricted KD group. It is important to note that the mice in this study were fed KetoCal, a new nutritionally balanced high fat/low carbohydrate ketogenic diet for children with epilepsy. This finding suggests that the use of KetoCal should be considered not only for epilepsy, but as an alternative therapeutic option for malignant brain cancer. Another study found that a KetoCal KD diet also increased mean survival time and slowed tumor growth in mice with brain cancer [149]. Additionally, one study on mice by Morsher et al. [150] compared a KD and SD on neuroblastoma, with or without calorie restriction. It was found that the best results were in the calorie restricted KD group, with reduced tumor growth and survival time. 

Meanwhile, a few studies have tried to compare the effect of a KD (with varying levels of carbohydrate amounts) on prostate cancer, with differing results. Caso et al. [151] studied mice that were either randomized into a standard Western diet, non-carbohydrate KD (NCKD) with 0% carbs, 10% carbohydrate KD, or 20% carbohydrate KD. The group with the slowest tumor growth was the 20% carbohydrate KD, while the WD had the most rapid growth. However, they did not find a significant improvement in survival among any of the carbohydrate restricted groups when compared to the WD. This result is different than a similar study done by Masko et al. [152], which compared a NCKD, 10% carbohydrate, and 20% carbohydrate diet in mice with prostate cancer. They concluded that none of these diet groups differed greatly in their tumor size throughout most of the study, and the diet did not affect survival. However, another study conducted on mice with prostate cancer compared a WD with a NCKD and found that the NCKD was significantly associated with lower tumor volumes at the end of the 53-day experiment [153]. Regardless of the varying results, a meta-analysis done by Klement et al. [154] analyzed a total of 29 animal studies and found that the majority (72%) found evidence of reduced tumor growth because of KDs. 

The data of the effect of KD in human patients is limited mostly to case studies and cohort studies. A meta-analysis of 24 human studies, found that 42% found that the KD can reduce tumor growth [154]. In addition, it has been found that most human studies had positive impacts [154,155], with many other studies found it stabilized disease [154,155] and one study found a pro-tumorigenic effect of the KD [154,155]. However, another review of 14 studies of the use of KD in cancer found mixed results [154]. It was found that people responded differently to the diet, with some cancers being reduced, some neutral in effect, and some cancers getting progressively worse. This finding could be related to a recent publication by Chang et al. [156] that tested relative expression of several key enzymes in ketolytic and glycolytic metabolism in human anaplastic glioma and glioblastoma. They found genetically heterogeneous tumors with varying expressions of key enzymes. However, they found most cells had an enzyme profile with decreased levels of mitochondrial ketolytic enzymes and increased expression of glycolytic enzymes, suggesting that human brain tumors are more dependent on glucose and have defects in ketone metabolism. 

The prognosis of patients with gliomas is extremely poor, with an average survival duration of 1.5 years [138]. Due to the poor outcomes with brain cancer, many studies using KD have been aimed at helping brain cancer patients. A small study by van der Louw et al. [157] followed three patients with recurrent diffuse intrinsic pontine glioma (DIPG). Although all three patients succumbed to the disease, it was determined that the use of KD is safe and feasible, but its effect on survival was not clear. Another 12-week randomized, controlled study also found that the use of KD in women with ovarian and endometrial cancer had favorable effects on physical function, perceived energy, and diminished food cravings for starchy and fast-food fats [158]. 

One of the most intriguing studies was a case study of a 38-year-old man with glioblastoma multiforme was treated with standard of care (SOC) along with a calorie-restricted ketogenic metabolic therapy, hyperbaric oxygen therapy, and other metabolic therapies [159]. The patient remains in excellent health with no neurological issues after 24 months of treatment. Thus, it seems that the ketogenic diet might be best utilized as an adjuvant therapy and should be started when the disease is first diagnosed. Recently the KEATING study [160] used either the modified ketogenic diet (MKD) or the medium chain triglyceride ketogenic diet (MCTKD) as an adjuvant therapy for glioblastoma. The Global Health Status (GHS) increased for patients in MKD cohort and decreased for the MCTKD patients. They had a low retainment with only 3 of 12 patients completing the 12-month intervention. The three patients who did complete the study chose to continue doing the KD. The researchers of the KEATING study suggested that the KD intervention should be reduced to six weeks and only be utilized during the time of chemo and radiation therapy. 

Yet, another study by Panhans et al. [161] had greater compliance. This study recruited patients with a diversity of CNS malignancies (GBM, astrocytoma, and oligodendroglioma). These patients were asked to do a more standard KD of 3:1 for 120 days and aimed to keep carbohydrates under 20 g/day. One cohort was provided KD meals by Epigenix Foundation for the first 30 days, while the others were given only meal plans. Adherence to the diet was confirmed with ketone and glucose levels measured with Precision Xtra meters. The six patients with the highest ketones were alive at the end of the study. The two patients with the lowest ketones succumbed to their disease. Five patients were able to maintain 100% adherence for the duration of the study. Overall, patients’ symptoms improved, which included higher energy levels, increased physical activity, increased cognitive function, decreased appetite, and reduced seizure. It is important to note that one patient had increased seizures. The researchers stated that the KD was well tolerated and discussed its feasibility for future experiments. This cancer clinic also stated that as interest in the KD grows, they now openly discuss the risks and potential benefits on a regular basis with patients and emphasize the lack of robust clinical evidence. 

The ketogenic diet is also now being tested as an adjuvant therapy for other cancers as well. For example, Clinicaltrials.gov currently lists over 100 trials looking at the ketogenic diet and 12 of those were related to CNS malignancies [161]. Therefore, data is starting to emerge on the impacts of KD on other cancer types. For instance, a study compared the typical diet with 55% of calories from carbohydrate (CHO) against a KD with around 6% from CHO in breast cancer patients in a 6-week trial [162]. The KD group’s global quality of life was higher at the 6-week mark and no adverse effects were seen in either group. Interestingly, the KD group lowered caloric intake without any restrictions, which may have been due to the satiating effects of fat. The KD diet was found to have no adverse effects on thyroid hormones, electrolytes, LDH, urea, or albumin. Yet, the KD diet was found to have potential beneficial effects, such as significantly reduced levels of lactate and ALP. Decreased lactate levels might slow metastases by reducing the acidity of the tumor microenvironment while reducing its ability to use it as a substrate for increasing biomass. Furthermore, it is believed that increased levels of ALP in breast cancer is a negative prognostic marker.

Another 12-week study in ovarian and endometrial cancer patients found an adherence level of 57–80% [163]. The focus of this study was to determine if the diet negatively affected lipid profiles since that is a current concern of many doctors and may restrict their decision on whether to suggest the KD diet for their cancer patients. They compared the KD versus the American Cancer Society (ACS) high-fiber, low fat diet. No changes were seen in lipid profiles to TC, TG, HDL-C, LDL-C, TC:HDL-C ratio or TG:HDL-C ratio after adjusting for baseline levels and weight loss. Another recent study looked at the effects of the diet on the body composition of KD patients while receiving radiation therapy. Klement et al. [164] compared a nonKD vs a KD with supplemental essential amino acids (KETOCOMP study). The KD had significantly associated with loss of 0.5 kg of fat mass and 0.4 kg of body weight per week, while showing no change in fat free mass or skeletal muscle mass. Thus, KD with ample amino acid intake could improve body composition during radiotherapy. Finally, a recent study conducted by Hagihara et al. [165] analyzed the effects of a 3-month KD as an adjuvant therapy for patients with advanced cancers of many types. They found that the diet was well tolerated, did not have any major negative outcomes, and improved life expectancy. Researchers were also able to stratify survival outcomes with three factors: albumin, blood sugar, and CRP levels. Thus, it was argued that stable adherence and highly reproducible results should be in favor of using the ketogenic diet as a standard for therapeutic treatment during chemotherapy with advanced cancer diagnoses. 

Please refer to Table S4 in the Supplementary Materials for a comparison of studies evaluating the KD in relation to cancer outcomes.

## 8. Discussion

A well-formulated ketogenic diet can provide low carbohydrate intake, while providing adequate fiber sources such as seeds, nuts, coconut, avocado, spinach, broccoli, cauliflower, and berries. Together, all these rich pre-biotic foods would lead to an increase in *Bacteroides* and *Bifidobacterium* and a subsequent decrease in *Firmicutes.* With disease rates increasing rapidly in the United States and other modern nations, it is increasingly important that we determine the safety, efficacy, and potential life-saving benefits of alternative diets. What might be discovered is that patients should be given individualized diets based on the species comprising their microbiome. This might enable patients to eat certain foods that maximize their ability to remain in a state of nutritional ketosis and optimize their overall health outcomes. The regular monitoring of the microbiome might be necessary to continually moderate and change dietary needs for diversity. It might also be determined that fecal microbiota transplants might be necessary to fully alter and change the microbiome at the onset of a new diet which could then be further modified and enhanced through diet. Regardless, much more research is needed in this area to determine the effect of the ketogenic diet on the microbiome.

Even though the ketogenic diet shows promise in helping patients lose weight, obesity is more than excess adipose tissue being stored on the body. It has been linked to many other metabolic issues, such as diabetes, cardiovascular disease, neurological disorders, and cancer. The ability to improve glycemic control in diabetics is critical for long-term health especially since some would argue that the biggest indicator of cardiovascular disease risk was HbA1c [88]. Surprisingly, the United Kingdom Prospective Diabetes Study (UKPDS) examined 5102 newly diagnosed type 2 diabetics and found that patients showed a 14% decrease in myocardial infarction for every 1% reduction in their HbA1c [166,167,168]. The ability to have tight glycemic control is even more challenging in type 1 diabetics since they are unable to make insulin and must inject it in response to glucose spikes induced by diet. Thus, their greatest challenge is controlling postprandial glycemia [98]. Some scientists argue that reducing carbohydrate intake is the easiest way for a type 1 diabetic to control their blood sugar levels since it will reduce the error in determining the insulin amount needed to match their increased blood glucose levels [88]. However, the benefits from the low carbohydrate diet might also improve other health markers in diabetics, such as abdominal fat and health-related quality of life factors as shown in other studies [169,170]. Type 2 diabetics have also improved or eliminated their diabetic state through diet, specifically a diet that restricts carbohydrate consumption. Type 2 diabetes results in insulin resistant cells and this has been linked to other complications and atherosclerotic processes such as inflammation, decreased size of LDL particles, and endothelial dysfunction [171]. Thus, the benefits of a healthy, low carbohydrate diet on diabetes might also improve the markers for cardiovascular disease as well.

Although the debate about diet and heart health continues, many new studies are revealing that the picture is much more complicated than the diet-heart hypothesis suggested. The need for more randomized, controlled studies of long-term duration are necessary to determine the true effect of dietary macronutrients on cardiovascular risks. It appears from preliminary studies that a ketogenic diet might have favorable outcomes on CVD, but some still view the idea with great skepticism. In medicine, randomized controlled trials are considered the gold standard and many physicians feel that there is not enough of these studies to consider changing their medical advice. It is interesting that while current scientists are unwilling to consider these dietary recommendations due to the lack of long-term evidence, the entire United States adopted the current dietary guidelines based mainly on an epidemiological study done by Ancel Keys [102]. Additionally, when the available randomized controlled studies and prospective cohort studies of that time were analyzed, they did not support the recommendation of dietary fat and coronary heart disease [172,173]. Regardless, the necessity in discovering a healthy diet for most people is an important endeavor, especially since we are currently seeing an epidemic of diabetes and obesity, both of which are linked to cardiovascular disease risk.

The potential of the ketogenic diet to aid in cancer treatment is still up for debate. However, the positive results seen in mice warrant that this metabolic therapy should be evaluated further. From the studies presented, it appears that in mice and humans, the diet seems to be most beneficial when used as an adjuvant with other therapies and when administered as soon as possible. It might also be critical to genetically analyze each tumor and determine its metabolic profile to determine if it is exhibiting the Warburg effect. If so, then the KD diet might be a useful addition to the treatment protocol. In summary, a ketogenic diet may have positive impacts on the pathogenesis of cancer, although the determination of its use as a monotherapy or adjuvant therapy in humans need further study. 

In conclusion, it is becoming more and more apparent that a “systems biology” approach to human health might be the way of the future. Future studies might need to consider numerous factors such as lifestyle, dietary intake, genotype, gut microbiome composition, and genome-wide information on the epigenome to create a successful plan for maximizing good health. According to Gerhauser et al. [20], “this ambitious goal can only be reached in large interdisciplinary research projects, combining expertise of food technologists, nutritionists, food chemists, molecular biologists, epigeneticists, clinicians, nutritional epidemiologists, bioinformaticians, and statisticians to achieve an integrated view of the influence of diet on human health.” Others argue that a diagnosis of high-risk epigenetic states may lead to a better understanding of the links between nutrition, the epigenome, and cancer risk [32]. If these markers can be identified and better understood, then new interventions can be created. New research suggests that long-term dietary choices affect diversity and gene expression of the gut microbiome. One such path might be the use of the ketogenic diet to increase beneficial metabolites which can have positive impacts on the genome. Additionally, a recent study analyzed the genetic variants for personalized management of ketogenic diets [174] and it suggested that certain genetic and dynamic markers of KD response may help identify individuals that will benefit the most from a KD diet. Thus, the use of the ketogenic diet might have a multitude of therapeutic effects, including but not limited to, helping with weight loss, improving lipid markers for cardiovascular health, healing a disrupted microbiome, improving epigenetic markers, reversing diabetes, or reducing the need for medication, and improving responses to cancer treatments. However, if a high fat/low carbohydrate KD diet seems too restrictive, then the use of personalized nutritional advice using microbiome sequencing might be the way of the future for stabilizing many of these diseases and improving metabolic health.

## Data Availability

Not applicable.

## Supplementary Materials

The following are available online at https://www.mdpi.com/article/10.3390/nu13051654/s1, Table S1: Main studies reporting the effects of low-carbohydrate diets, including ketogenic diets, on weight loss, Table S2: Main studies reporting the effects of low-carbohydrate diets, including ketogenic diets, on diabetes health markers, Table S3: Main studies reporting the effects of low-carbohydrate diets, including ketogenic diets, on lipidology health markers, Table S4: Main studies reporting the effects of low-carbohydrate diets, including ketogenic diets, on cancer.
