# Supplementary material for: The Potential Health Benefits of the Ketogenic Diet: A Narrative Review
Source: Nutrients. 2021 May 13;13(5):1654. doi: 10.3390/nu13051654 (PMC8153354; doi:10.3390/nu13051654)
Supplement: Supplementary file 1 [file nutrients-13-01654-s001.zip › nutrients-1193881-supplementary.pdf]

Table S1: Main studies reporting the effects of low-carbohydrate diets, including ketogenic diets, on weight loss.

References	Sample size	Type of diet	Health Status	Duration	Weight loss outcomes
Athinarayanan 2019	N = 262	KD	T2	2 year follow-up	-10% Mean Body weight reduction<br>Reductions in abdominal fat
Bruci 2020	N = 92	Very low-calorie VLCKD	Obesity and mild kidney failure	3 months	Average weight loss ~20% initial weight<br>Decreased fat mass (-11.23 kg)<br>Decreased BMI (-5.64 kg/m2)
Carmen 2020	N =3	KD	Obesity<br>Comorbid binge eating food addiction	6-7 months	Reduced body weight (10-24%)<br>Weight maintenance for 9-17 post treatment with continued adherence Decreased BMI (3.8-6.8 kg/m2)
Castro 2018	N =20	Very low-calorie VLCKD	Obese	4 month	Decreased BMI (7.7)<br>Decreased fat mass (18 kg)<br>Decreased visceral fat (1.2 kg)
Choi 2018	N = 46	KD 4:1 and KD 1.7:1	Obese Adults	2 weeks	Decreased waist circumference<br>Decreased BMI<br>Decreased Body fat mass<br>Decreased Body weight<br>Skeletal Mass Unchanged only in 1.7:1 KD
D'Abbondanza 2020	N = 70	Very low-carbohydrate VLCKD	Severe Obesity	25 days	Reduced waist circumference (Males -8.5 cm vs Females 6.0 cm)<br>Decreased BW (Males -13 kg vs Females -10kg)<br>Decreased BMI (4 kg/m2)<br>Decreased Fat mass (Males -4% vs Females -3%)
Dashti 2007	N=64	KD	T2<br>Obese	56 weeks	Significant loss in BW
Dashti 2006	N =66	KD	Obese High Cholesterol	56 weeks	Decreased BW (-25.9 %)<br>Decreased BMI
Ebbeling 2018	N = 164	Low carb diet (20% carb)	Adults BMI >25	20 weeks	Total energy expenditure increased 52kcal/d for every 10% decrease in carbohydrate contribution to total energy intake.
Gibas 2017	N = 30	Benign dietary ketosis	Metabolic syndrome	10 weeks	Decreased BW<br>Decreased BMI<br>Increased RMR
Gomez-Arbelaez 2018	N = 20	Very low calorie KD	Obese	4 months	Fat Mass Decreased -16.5 kg<br>Minor Reduction in Fat Free Mass -3.8kg<br>RMR unchanged

Goss 2020	N = 34	Very low carb diet VLCD	Obese	8 weeks	9.7% Total Fat loss<br>-22.8% VAT loss<br>Decreased BW
Guldbrand 2012	N = 61	Low carb Diet (20% carb)	T2	24 months	Weight loss (-4.31 kg)
Hall 2016	N=17	KD	Overweight or obese	8 weeks	Increased Energy expenditure<br>Weight loss in 4 week KD (-2.2 kg)<br>Body fat loss (0.5kg)
Hallberg 2018	N = 349	KD	T2	1 year	Decreased BW (Average -13.8 kg)
Hussain 2012	N = 363	Low carb KD	Obese<br>T2	24 week	~12% Weight loss<br>Decreased waist circumference (-6-7%)
Lennerz 2018	N = 316	Very low carbohydrate diet (VLCD)	T1	At least 3 months	None
Kong 2020	N=20	Shortterm KD	Obese Chinese females	8 weeks	Reduced body weight (-2.9kg)<br>Reduced BMI (-1.1kg)<br>Reduced waist circumference (-4.0cm)<br>Reduced hip circumference (-2.5cm)<br>Reduced body fat (-2.0%)<br>Reduced fasting leptin levels
Martin 2011	N = 270	Low carb diet LCD (<20g/d)	Obese	2 years	Decreased cravings for carbs<br>Decreased BW (-7.8%)<br>Decreased hunger
McClernan 2007	N =119	Low carb KD	Overweight	6 months	Decreased BW (-12.9 kg)<br>Decreased hunger
Michalzyk 2020	N = 91	Low calorie KD	Obese overweight	12 week	Decreased BW (-13.72 kg)<br>Decreased BMI (8.05 kg/m2)<br>Decreased Waist circumference (-13.7 cm)<br>Decreased Hip circumference (-11.61 cm)<br>Decreased thigh circumference (-7.66)
Mohorko 2018	N = 35	KD + short term calorie restriction	Sedentary obese adults	12 weeks	Male weight loss (-18kg)<br>Female weight loss (-11kg)<br>Significant loss in fat mass (Male:-6kg and Females -3kg)<br>Reduced leptin levels<br>Slight increase energy expenditure
Monda 2020	N = 20	Very low calorie KD	Obese	8 weeks	Decreased BW (-12.6 kg)<br>Decreased BMI (-4.43 kg/m2)<br>Decreased VAT (-613 g)

					Decreased FM (-11,831 g)<br>Fat free mass and bone mineral density unchanged
Moreno 2016	N = 45	Very low calorie KD followed by transition to balanced diet	Obese	24 months	Decreased BW (-12.5 kg)<br>Decreased BFM (-8.8 kg)<br>Decreased WC (-11.6 cm)<br>Decreased VAT
Samaha 2003	N = 132	Low carb diet (<30g/day)	Severely obese	6 months	Weight loss -5.8 kg
Shai 2008	N = 322	Low carbohydrate, non-restricted calorie (20-120 g/day)	Moderately obese	2 years	-4.7 kg overall weight change<br>Mean weight change -5.5kg<br>Mean BMI -1.5<br>Significant decrease waist circumference
Shih 2019	N =609	Healthy low carb HLC	Generally healthy with BMI 28-40 kg/m2	12 months	Average decrease body weight (-6.3 kg)<br>As percentage of SFA increased, body weight lost increased
Walton 2019	N = 11	Low carbohydrate KD	T2	90 days	Average weight loss -9kg

Table S2: Main studies reporting the effects of low-carbohydrate diets, including ketogenic diets, on diabetes health markers.

References	Sample size	Type of diet	Health Status	Duration	Diabetic Outcomes
Ahmed 2020	N =49	LCHF	T2	≥ 3 months	-1.29% in A1C<br>-43.5 mg/ml reduction in fasting BG<br>100% on insulin discontinued or reduced insulin dose.
Athinarayanan 2019	N = 262	KD	T2	2 year followup	Glycemic medication declined from 55.7 to 36.8%<br>-62% in insulin<br>53.5% diabetes reversal<br>17.6% diabetes remission
Bruci 2020	N =92	Very low-calorie ketogenic diet (VLCKD)	Obese	3 months	Significant reduction in fasting glucose and HbA1c
D'Abbondanza 2020	N = 70	Very low-carbohydrate VLCKD	Severe Obesity	25 days	Reduced HbA1c (Males -0.6% vs -1.4%)<br>Reduced HOMA-IR (Males -6.8 vs Females -2.6)
Dashti 2006	N=66	KD	Obese<br>High cholesterol	56 weeks	Decreased blood glucose (-31%)
Dashti 2007	N=64	KD	T2<br>Obese	56 weeks	Significant decrease in blood glucose of diabetic patients (10.5 mmol/l to 5 mmol/l)
Gibas 2017	N = 30	Benign dietary ketosis	Metabolic syndrome	10 weeks	Decreased HbA1c
Goss 2020	N=34	Very low carbohydrate diet (VLCD)	Obese	8 weeks	Significant decrease in fasting insulin<br>Decreased HOMA-IR<br>Increased insulin sensitivity
Hallberg 2018	N = 349	KD	T2	1 year	-1.3 HbA1c<br>94% insulin therapy reduced or eliminated
Hussain 2012	N = 363	Low carb KD	102 patients with T2	24 week	HBA1c decrease (7.8 to ~6.3)<br>Decrease BG (9.1 mmol/l to ~6.4)
Krebs 2016	N = 10	Low carb (<75g/day)	T1	12 week	HbA1c decrease (63 to 55 mmol/mol)<br>Reduced insulin (64.4 to 44.2 units)
Lennerz 2018	N = 316	Very low carbohydrate diet (VLCD)	T1	At least 3 months	Mean HbA1c is 5.67%<br>Mean daily insulin dose 0.40 U/kg per day
Lichtash 2020	N =1 case study	KD and intermittent fasting	T2	14 months	HbA1c reached 5.8%

Michalczyk 2020	N = 91	Low calorie KD	Hyperinsulemic and hyperglycaemic	12 week	HbA1c decreased (5.87 mg/L to 5.38)<br>Glucose decreased (5.94 mmol/L to 4.74)<br>Insulin decreased (14.12 ug/Dl to 6.61)<br>HOMA-IR decreased
Mohorko 2018	N = 35	KD + short term calorie restriction	Sedentary obese adults	12 weeks	Significant decrease in serum glucose followed by return to baseline<br>Insulin levels decreased continually
Monda 2020	N = 20	Very low calorie KD	Obese	8 weeks	Decreased glycemia<br>Decreased HbA1c<br>Decreased insulinemia
Neilsen 2012	N =48	Low carb (<75g/day)	T1	4 year follow-up	Adherent dieters achieved HbA1c decrease (7.8 to 6.0%)
Saslow 2017	N =34	Very low carb KD	T2 or prediabetes	12 months	HbA1c decrease (6.6 to 6.1%)<br>Reduction in diabetes medications
Saslow 2017	N =25	Very low carb KD	T2	32 weeks	More than half patients lowered HbA1c to <6.5%
Shai 2008	N = 322	Low carbohydrate, non-restricted calorie (20-120 g/day)	Moderately obese	2 years	Significant decrease in HbA1c (-0.9)
Tay 2015	N = 115	Very low carbohydrate, high unsaturated fat	T2	54 weeks	Reduced HbA1c (-1.0%)<br>Reduced fasting glucose (0.7mmol/L)<br>Greater reduction in in diabetes medications
Walton 2019	N = 11	Low carbohydrate KD	T2	90 days	HbA1c decreased to 5.6%
Webster 2019	N = 24	LCHF	T2	15 months at follow-up	HbA1c decreased from 7.5 to 5.9%
Westman 2008	N = 84	LCKD	T2	24 weeks	HbA1c decreased 1.5%<br>95.2% reduced or eliminated diabetes medications
Wong 2020	N = 14	KD	T2 or T1	≥ 3 months	Improved blood glucose levels, reduced or stopped diabetes medications

Table S3: Main studies reporting the effects of low-carbohydrate diets, including ketogenic diets, on lipidology health markers.

References	Sample size	Type of diet	Health Status	Duration	Lipid Outcomes
Athinarayanan 2019	N = 262	KD	T2	2 year followup	Decreases in systolic and diastolic blood pressures<br>Decreased triglycerides<br>Increased HDL<br>Increased LDL (decrease sdLDL and large VLDL-P, increase in LDL particle size partitioning with no change in ApoB)<br>No change in TC
Bruci 2020	N = 92	Very low-calorie VLCKD	Obesity and mild kidney failure	3 months	Decreases in systolic and diastolic blood pressures<br>33.3% on antihypertensive medications reduced or eliminated<br>Reduced TC and TG<br>No change in HDL or LDL
Choi 2018	N = 46	KD 4:1 and KD 1.7:1	Obese Adults	2 weeks	Total Cholesterol Unchanged (4:1) or Decreased (1.7:1)<br>TG unchanged<br>LDL Unchanged (4:1 KD)<br>LDL Decreased (1.7:1)<br>HDL unchanged
Dashti 2007	N=64	KD	T2<br>Obese	56 weeks	Decreased in LDL, Total cholesterol, and TG<br>Increase in HDL/LDL ratio<br>Increase in HDL
Dashti 2006	N=66	KD	Obese<br>High cholesterol	56 weeks	Decreased in LDL (-28.2%),<br>Decreased Total cholesterol (-19.3%)<br>Decreased TG (-59%)<br>Increase in HDL (+52.3%)
Ebbeling 2018	N = 164	Low carb diet (20% carb)	Adults BMI >25	20 weeks	Decreased TG<br>Increased HDL
Gibas 2017	N = 30	Benign dietary ketosis	Metabolic syndrome	10 weeks	Decrease in TG
Goss 2020	N=34	Very low carbohydrate diet (VLCD)	Obese	8 weeks	Increased HDL<br>Decreased TG<br>TC and LDL unchanged

Guldbrand 2012	N = 61	Low carb Diet (20% carb)	T2	24 months	Increased HDL<br>LDL unchanged
Hall 2016	N=17	KD	Overweight or obese	8 weeks	Decrease in TG (Ave 85.4 mg/dL)
Hallberg 2018	N = 349	KD	T2	1 year	Decreases in systolic and diastolic blood pressures<br>Decreased triglycerides (-24%)<br>Increased HDL (+18%)<br>Increased LDL (+10%)<br>No change in ApoB)
Hussain 2012	N = 363	Low carb KD	102 patients with T2	24 week	Decreased TG<br>Decreased Total Cholesterol<br>Increased HDL<br>Decreased LDL
Lennerz 2018	N = 316	Very low carbohydrate diet (VLCD)	T1	At least 3 months	Low TG<br>Elevated HDLc<br>High Total cholesterol<br>High LDLc
Michalzyk 2020	N = 91	Low calorie KD	Obese overweight	12 week	Decreased TG (-84.32 mg/dL)<br>Increased HDL (16.28)
Mohorko 2018	N = 35	KD + short term calorie restriction	Sedentary obese adults	12 weeks	Temporary increase in LDL (2 weeks) then returned to baseline
Monda 2020	N = 20	Very low calorie KD	Obese	8 weeks	Decreased TC<br>Decreased LDL<br>Decreased TG<br>HDL unchanged
Norwitz 2020	N =1	HFLC KD	Inflammatory Bowel disease	7 months	Increased TC (160 to 450 mg/dL)<br>Increased LDL-C (95 to 321 mg/dL)<br>Increased LDL-P (1,143 to 2,259; Small & Medium decreased and Large (ApoB) Increased)<br>Decreased LDL<br>Increased HDL-C (48 to 109 mg/dL)<br>Increased HDL-P (5,699 nmol/L to 12,080)<br>CAC score 0
Samaha 2003	N = 132	Low carb diet (<30g/day)	Severely obese	6 months	Decrease TG (-20%)<br>TC, HDL, and LDL unchanged

Shai 2008	N = 322	Low carbohydrate, non-restricted calorie (20-120 g/day)	Moderately obese	2 years	Significant decrease in blood pressure<br>(Systolic -3.9 mmHg and -08 in diastolic)<br>Decrease in TG (23.7 mg/dL)<br>LDL unchanged<br>Increased HDL (8.4 mg/dL)<br>20% decrease in TC/HDL
Shih 2019	N =609	Healthy low carb HLC	Generally healthy with BMI 28-40 kg/m2	12 months	Higher LDL (119.8)<br>Higher HDL (53.1<br>Lower TG (100.6)<br>Every 1% increase in SFA intake resulted in decrease in TG without changes in LDL or HDL
Walton 2019	N = 11	Low carbohydrate KD	T2	90 days	Lower systolic and diastolic blood pressure (-10.7 and -7.3 mmHg)<br>Increased HDL (+9.2 mg/dl)<br>Decreased TG (-84.9 mg/dl)<br>LDL unchanged<br>Decreased TG:HDL ratio (-2.8)

Table S4: Main studies reporting the effects of low-carbohydrate diets, including ketogenic diets, on cancer.

References	Study Type	Type of diet	Cancer Type	Outcomes
Abdelwahab 2012	Mice	KetoCal KD	Brain	Decreased Tumor Growth<br>Increased Survival Time
Allen 2013	Mice	KD	Lung	Decreased Tumor Growth
Caso 2013	Mice	KD (0% CHO, 10% CHO, or 20% CHO)	Prostate	Slowest Tumor Growth in 20% CHO group<br>Mean Survival Time Unchanged
Cohen 2018	Human Women	KD	Ovaria and endometrial	Favorable effects on physical function, perceived energy, and diminished food cravings for starcy and fast foods.
Cohen 2020	Human	KD	Ovarian and endometrial	57-80% diet compliance<br>No negative impact on lipid profiles
Elsakka 2018	Case study of man	Calorie-restricted KD & hyperbaric oxygen therapy	Glioblastoma multiforme	Excellent health with no neurological issues after 2 years of treatment.
Hagihara 2020	Human	KD	Advanced cancers	Improved life expectancy
Khodabakshi 2020	Human	KD	Breast	Positive outcomes: Lowered caloric intake, Significantly reduced lactate and ALP

Kim 2012	Mice	KD (0% CHO)	Prostate	Lower Tumor Volumes
Klement 2017	Meta analysis of 29 animal studies	KD	Varying cancer types	72% Had Decreased Tumor Growth
Klement 2017	Meta-analysis of 24 human studies	KD	Varying cancer types	42% had Decreased Tumor Growth
Klement 2019	Human	KD	Varying cancer types	Improved body composition while receiving radiotherapy
Martin-McGill 2020	Human	Modified KD	Glioblastoma	Global Health Status increased
Masko 2010	Mice	KD (0% CHO, 10% CHO, or 20% CHO)	Prostate	No difference in tumor growth<br>No effect on survival time
Morsher 2015	Mice	Calorie restricted KD	Neuroblastoma	Decreased Tumor Growth<br>Increased Survival Time
Oliveira 2018	Meta-analysis of 14 human studies	KD	Varying cancer types	Some positive outcomes, some neutral outcomes, and some did worse.
Otto 2008	Mice	KD	Gastric	Decreased Tumor Growth<br>Increased Survival Time
Panhans 2020	Human	KD	CNS malignancies	Increased survival time for those with highest diet compliance and high ketone levels. Increased energy levels, increased physical activity, increased cognitive function, decreased appetite, and reduced seizures.
Poff 2013	Mice	KD	Systematic Metastatic	Lowered Blood Glucose<br>Decreased Tumor Growth<br>Increased Survival Time by 56.7%
Van der Louw 2019	Human	KD	Recurrent diffuse intrinsic pontine glioma (DIPG)	Safe and feasible, but effect on surivival unclear
Zhou 2007	Mice Human	KetoCal KD	Malignant Mouse Astrocytoma & Human Malignant Glioma	Decreased Tumor Growth by 65%<br>Reduced angiogenesis
